# Support needed by nursing students to develop professional dignity

**DOI:** 10.1177/09697330251328688

**Published:** 2025-04-13

**Authors:** Isabel Hlupheka Shilenge, Neltjie Christina van Wyk, Anna Elizabeth van der Wath

**Affiliations:** 56410 University of Pretoria

**Keywords:** Nursing students, professional dignity, professional growth, professional nurses, support, work-integrated learning

## Abstract

**Background:**

Nursing students’ professional dignity development during work-integrated learning is dependent on support from professional nurses. If they are left unsupported, such development is jeopardised.

**Aim:**

The aim of the study was to explore and describe the support that nursing students need from professional nurses, including their lecturers, during work-integrated learning to develop professional dignity.

**Research design:**

A qualitative, exploratory-descriptive research design applied. Through volunteer sampling, participants were invited for face-to-face in-depth individual interviews to discuss the question: ‘What support did you need from others during work-integrated learning to feel dignified as nursing students?’ Saturation of data determined the number of participants. Recording of the interviews and the writing of field notes were carried out with the permission of the participants. Manual coding in a thematic analysis was done to analyse the intricate data content with intuition and insight.

**Participants and research context:**

The study was undertaken in South Africa at a designated nursing education institution and the hospital where the students performed the bulk of work-integrated learning. Fourteen third-year students were interviewed. They had sufficient experience with work-integrated learning and could provide rich data concerning support needed to develop professional dignity.

**Ethical considerations:**

The Faculty of Health Sciences Research Ethics Committee at the University of Pretoria approved the proposal (Reference number 73/2023) and the applicable authorities gave written permission for the research to be conducted. Since the participants were students, the researchers made sure that they did not feel obliged to participate.

**Findings:**

Four categories were identified, namely, (a) improving work-integrated learning experience, (b) value students’ professional development, (c) cooperate to benefit students’ professional growth and (d) manage resources optimally.

**Conclusion:**

Students needed to be respected and their input to quality nursing care acknowledged. Positive role models and learning conducive clinical environments contributed to their development.

## Introduction and background

Nurses’ professional dignity is determined by the way patients and members of the health team perceive their capabilities. When they are trusted to perform their duties and respected for their contribution to quality patient care, their professional dignity is confirmed. Their own perception of their value in patient care contributes to their sense of professional dignity.^1^

Nursing students are trained to uphold the dignity of patients^2^ and are guided by lecturers to respect the dignity of members of the health team.^3^ Much time is required to enable them to develop their own professional dignity in interaction with others during clinical and theoretical training.^4^ According to Stikholmen, Nåden and Alvsvåg^5^ exposure to people from different social and cultural backgrounds may enhance students’ professional dignity development. Students, however, require guidance from lectures and clinical facilitators to identify with the nursing profession.^6^ They need caring learning environments to establish professional images.^5^ When they feel empowered to perform tasks on their level of training, they easily develop positive self-esteems and a sense of professional dignity.^7^

It is unfortunate that students often experience clinical situations that threaten their professional growth^8^ with detrimental effect on professional dignity development.^9,10^ When they feel insecure during work-integrated learning,^5^ are bullied by senior students^8^ and treated unfairly by supervisors^10^ their professional dignity development is jeopardised resulting in poor performances in professional growth.

At the hospital associated with the designated nursing education institution, nursing students were at times left unsupported and unguided during work-integrated learning. The students also voiced their concerns about being treated by the health team disrespectfully. They often endured belittlement to the detriment of their professional development.^11^

Research is often focussed on support of patients’ dignity^1,3^ and the professional dignity of nurses and midwives.^12,13^ Humiliation and demanding work environments often pose threats to nurses’ dignity^12^ while their dignity gets enhanced when their capabilities are acknowledged, their interventions are appreciated, they are perceived as equal health team members and they work in conducive environments.^13^

This study aimed at exploring the support that the nursing students needed to develop professionally. The purpose of the study therefore referred to the respect that the students wished the professional nurses who supervised them during work-integrated learning could have for them as future professional nurses. It also refers to the willingness of the professional nurses to assist students to develop professionally.

## Setting

The study was undertaken in South Africa at a designated nursing education institution and the tertiary care hospital where the students performed the bulk of work-integrated learning. The institution had at the time of the study 290 students registered for a 3-year diploma programme in nursing. The students were placed at the hospital for 24 weeks spread according to the content of the subject modules, working a 40-h week under supervision of profession nurses. Thirty-eight lecturers facilitated both theory classes and did clinical accompaniment of the students during practical placements. During work-integrated learning professional nurses employed by the hospital also supervised the students. The designated hospital had 857 beds, with an average of 506 professional nurses taking care of patients.

## Methods

An exploratory-descriptive qualitative study through individual interviews was done to gain data about the support that students needed to develop senses of professional dignity. In qualitative studies, participants are encouraged to share their opinions in their own words about events that they personally experienced. The design suited the study as the students were given the opportunity to describe the support that they wished the professional nurses could have given them during work-integrated learning. For this study, 97 third-level nursing students registered for the diploma programme composed the study population. They all met the inclusion criterium of having completed work-integrated learning. Volunteer sampling applied. Once the first author got approval from the Research Ethics Committee and the nursing institution management gave permission for the research to be conducted, she explained the planned research to the students and requested volunteers for the sample. Fourteen students were interviewed before data saturation occurred. No new data were gathered during the interview with the thirteenth participant. One extra participant was interviewed to ensure that data saturation was reached.

Data collection was conducted through face-to-face in-depth individual interviews in a venue accessible to the participants. Privacy was maintained and all the participants gave informed consent to take part in the interviews and that it could be audio-recorded. The first author conducted the interviews, using only one question: ‘*What support do you need from others during work-integrated learning to feel dignified as a nursing student*?’ and probes such as ‘Can you tell me a little more?’ to get a rich description of the data. Theoretical notes were written to make sense of the participants’ descriptions and reflective notes to document personal experiences and reflections, before, during and after the interview in an effort to prevent biasness.^14^

A thematic data analysis as described by Liamputtong^15^ was done. The first author familiarised herself with the data by listening to the audio recordings repeatedly and reading and re-reading the transcripts and fieldnotes to gain an understanding of the participants’ views. Manual coding was done to analyse the intricate data with intuition and insight. Codes were identified and grouped according to related content. The codes were arranged to form sub-categories and similar sub-categories formed categories. The second author followed the same steps and validated the sub-categories and categories that the first author identified.

## Ethical considerations

Before the commencement of the research, the Faculty of Health Sciences Research Ethics Committee at the University of Pretoria approved the proposal (Reference number 73/2023) and the applicable authorities gave written permission for the research to be conducted at the designated institution. Since the participants were students, the first author who was a lecturer at the designated institution, made sure that they did not feel obliged to take part in the study. Their privacy and the confidentiality of the information that they shared were ensured. Codes were used to keep the participants’ identities confidential.

## Trustworthiness of the findings

Rigour of the findings was ensured with the use of the strategies of credibility, transferability, dependability and confirmability.^16,17^ The credibility of the findings was confirmed through member checking and peer debriefing. The data analysis process and the outcome of the process was discussed with participants (for member checking) and the research supervisors of the first author (for peer debriefing) to ensure that the findings represented the data obtained from the participants. The participants agreed with the accuracy of the interpretation of the data and the supervisors confirmed the applicability of the identified categories and sub-categories. Fourteen participants were interviewed before the data collection was terminated due to saturation of data. The interviews provided sufficient data to enable a thorough understanding and description of the studied phenomenon.

The transferability of findings refers to the applicability of it to similar contexts with similar participants.^18^ In this study, a thorough description of the participants and the context in which the data were collected enable educators to decide whether the findings can be applied to help their students to develop professional dignity.

The dependability of the findings was ensured. The comprehensive description of participant sampling, data collection and the analysis of the data created an audit trail to be used in the replication of the research. The researchers confirm that should the research be repeated in the same manner similar results are possible. Excerpts from the transcripts of the interviews were used to substantiate the description of the sub-categories in order to ensure the confirmability of the findings. The categories and sub-categories represent the data obtained from the participants.^19^

## Demographic data of the participants

Data were collected from September to December 2023, with student participants in their third level of study at the designated institution. The sample comprised 14 participants (11 females and 3 males). The ages of the participants ranged from 21 to 30 years, and all were Black African students, but from different ethnicities (refer to Table 1). The participants were from rural, semi-urban and urban areas in the Gauteng Province of South Africa and completed their secondary education at public schools in the vicinity of the designated nursing education institution.

**Table 1.** Demographic data of the participants.

Participants	Age group	Gender
P1	31–35	Female
P2	20–25	Female
P3	26–30	Female
P4	31–35	Female
P5	20–25	Female
P6	31–35	Female
P7	26–30	Male
P8	26–30	Female
P9	26–30	Female
P10	20–25	Female
P11	26–30	Female
P12	31–35	Male
P13	26–30	Male
P14	26–30	Female

**Note:** P = Participant number.

## Findings

The student participants wished that improved learning experiences, acknowledgement of their contribution to patient care and their involvement in decision-making in the clinical environment could contribute to their development of professional dignity (refer to Table 2 for the categories and sub-categories).

**Table 2.** Categories and sub-categories of the study.

Categories	Sub-categories
Improving work-integrated learning experience	Provide guidance in clinical learning
	Correlate tasks with learning objectives
	Initiate learning opportunities
	Create learning conducive environment
	Enable theory-practice integration
	Role model professional behaviour
	Enhance teamwork and collaboration
Value students’ professional development	Acknowledge students’ personal beliefs
	Respect students’ contribution to patient care
	Advocate for students’ dignity
	Be patient with students’ development
	Enable psychological support
Cooperate to benefit students’ professional growth	Enhance NEI/clinical practice cooperation
	Enable information sharing
Manage resources optimally	Increase time clinical lecturers spent with students
	Secure sufficient time for students’ development

## Category: Improving work-integrated learning experience

The participants wanted to be guided on how to perform nursing procedures. They wished that professional nurses would supervise them when they perform nursing tasks. Without proper guidance, they often felt lost and worthless:

“… with professional nurses, I think for them would be to just hold our hands. We are not perfect. We are new. There’s lot of things we don’t know, and I said we don’t have a lot of time in facilities…so, a lot of things we are clueless…” (P4)

“…for them to at least show us the correct way of doing procedures um and how to handle certain situations because I’m going to be a professional nurse which means I have other people that I have to be responsible for, so they also teach us those types of responsibilities and supervise us so that we are ready to act in their footsteps...” (P10)

The participants complained that they were assigned tasks during work-integrated learning that were not included in their learning objectives to the detriment of their professional development:

“…It makes me feel like I am not growing as a professional person, am I being taken serious by the people who are there or they just see me as a student, and I’m just there to perform what they want me to do?” (P9)

“…We were forced to do the work that is not meeting our objectives, so even though the units in the hospitals receive our objectives, they still do not regard them; they only want to assist us after we have done their work…” (P14)

The participants were often not exposed to clinical learning opportunities. They wanted the professional nurses who supervised them during work-integrated learning to initiate opportunities for them to develop their clinical skills:

“…Professional nurses can give us the platform to be able to work, to be able to learn by showing us some of the things we don’t know, by showing us some of the things that we are unable to do…by doing so, we can get…the necessary confidence, we can build…our self-esteem…” (P7)

“…In most cases, we are not being involved in the decision-making part of things that are done in the healthcare facilities…we are just there; they give you instructions to do whatever…I would like for them to involve us throughout the nursing process…” (P5)

“…So, I think for me…as a student in such a situation, I would like to be part of meetings because you get to see…how we may…work together…” (P3)

“…So, involve them in the…activities that it’s in the ward…cause usually what you see, you will find students being excused whenever there is a meeting, excused whenever there is… resuscitation…when are they going to learn these things…” (P6)

When the professional nurses created clinical environments conducive for the students to gain clinical knowledge and skills, the participants’ sense of professional dignity were stimulated:

“…So, it should be an environment such that it allows for me to grow and learn and to build confidence in my skills…” (P1)

The participants complained that the professional nurses in the wards poorly implemented theory and practice integration. The situation hindered their skills development in using theory in improving patient care. What they had been taught in theoretical classes did not correlate with the nursing care that they observed in the wards:

“…You will report a vital sign that is abnormal, you will say ‘blood pressure of Mrs So-and-So is deranged’, and then the sister will say ‘ai, no don’t worry, it’s always like that’, and the student is somehow conflicted to say’ but I am told at the college, to report it, I get the opposite. The sister is brushing it off, no one is saying anything, like the abnormal is normal…it’s…confusing…” (P6)

Unfortunately, not all professional nurses were proud of their profession. Some complained about being nurses. The participants hoped that the opposite was true. They wanted the professional nurses to be positive role models to them:

“…if they show us that they are happy to be in the profession then…it’s nice to be there too…I think I would also follow in their footsteps…that give us hope… make us proud of being in this profession…acting correctly and doing things according to the law...” (P5)

The student participants wanted the professional nurses to make them feel welcome in the clinical nursing teams. They believed that if they have been allowed to take part in team work, they could have developed a sense of professional dignity:

“…you know if somebody makes you feel like you are not part of the team, it side lines you, it puts you in a corner…and being put in a corner makes you question your abilities to actually perform a task…if you are not resilient enough, then it just might demoralise you…you don’t want to be there…it downgrades you, you know it does not contribute to professional dignity…” (P11)

## Category: Value students’ professional development

The participants’ input to patient care was not valued and their development to become professional nurses was not acknowledged:

“…because most of the time we get treated as though our opinions don’t matter, so you get told, you get spoken onto, it’s not on equal basis.” (P1)

“…I would appreciate it if the multidisciplinary team recognised students, nursing students particularly…as people who are competent in what they are there for, and who are also able to execute tasks...” (P6)

When lecturers ensure that students are exposed to clinical learning opportunities and they are not misused to perform routine care, their professional growth is supported:

“…We need advocacy from our lecturers. We need transparency also such that when we get there (in the wards) …maybe an in-service training with the staff to alert everyone that when students come, they are not extra hands to assist them to lessen their workload…they have come to learn…” (P1)

The participants felt insecure when they had to adjust to differing clinical environments. They needed professional nurses to explain to the staff of the designated ward that they were students who required guidance from experienced nurses. A participant described it as follows:

“…We are not qualified, we are not perfect, that’s why we need that 30 minutes to do certain skill that professional nurses can do in 5 minutes. If they can also be lenient with us…be patient with us until we learn, so that also we can be able to teach others as well…” (P4)

A participant reiterated that the students needed psychological support to transition into becoming professional nurses:

“…Being a nursing student comes with a lot of strain, it comes with a lot of emotional distress… also…a lot of responsibility, you are given a lot of responsibility, and being taught how to work under minimum supervision…carries own responsibilities as well…that for me says that there should be a lot of psychological support…” (P11)

## Category: Cooperate to benefit students’ professional growth

The participants complained about poor communication between the designated nursing education institution and the hospital where they did work-integrated learning. The nursing procedures demonstrated at the institution differed from the procedures that were used in the hospital. It caused the participants to feel insecure and they therefore struggled to develop professional dignity:

“…These are the new outcomes that we are supposed to learn…I feel like those things are supposed to be communicated to the professional nurses and the institutions as well. I think they should…the clinical lecturers and the theory lecturers at the college and the professional nurses at the hospitals and clinics should sit down and actually teach each other the different procedures…they teach them about the programme that is being introduced in the different colleges, and they are able to collaborate...” (P2)

Acknowledging that students are a part of multidisciplinary healthcare teams may help them to develop their professional dignity. Involving them in the team when patient care is discussed, may help them to grow professionally:

“…If we have questions for them that we’d like to direct straight to the doctors and then not go through nurses. I would like for them to help us with the information…I would like to think they have more information than we do so I would like for them to engage us...” (P5)

## Category: Manage resources optimally

The participants wished that more lecturers could have been available to help them to develop their professional dignity during work-integrated learning:

“…At the moment they’re coming once a week and by the time they come once a week, you cannot learn something in one day actually…so, I feel like if they could come maybe I don’t know how many times a week, so that they can show us some of the procedures because some of the procedures are critical in nursing and once you miss them you can never go back…” (P7)

Limited time was available for work-integrated learning. The participants would like the curriculum to be changed to allow more time for this type of learning. According to them less time should be spent in lecture rooms and more time in the clinical environment:

“…I am not sure if that’s the curriculum design…but remember the time allocation, different aspects will require different time allocation…you can’t just allocate 1 hour for the specific module that requires that will be covering 2 topics and then allocate the same hour for a different module that needs to cover 6 topics…” (P9)

## Discussion

During work-integrated learning students are supervised by professional nurses to gain knowledge and skills in nursing science and to develop into professional nurses.^20,21^ With sufficient support during work-integrated learning, students develop a sense of professional dignity. According to the input of the participants of this study much can go wrong during work-integrated learning. Not all professional nurses serve as role models for students’ professional growth. According to Keis et al.^22^ students learn their professional behaviour from working with and observing professional nurses when they perform nursing procedures. Students learn from what they see, hear and observe^23^ and should they be exposed to negative behaviour of professional nurses, it may be very challenging for them to grow professionally and to function with dignity. Positive role modelling may stimulate and enforce students’ professional identity and dignity development.^24^

When students are expected to perform routine nursing care and their learning objectives are neglected their professional development is jeopardised. The participants complained that it often happened that they could not practice procedures expected of third-year students. Such situations can be viewed as student bullying^10^ as their professional development gets strained. Affording students opportunities to develop the competencies expected of them in their learning objectives may enhance their confidence and assure their dignity development.^25^ Trust in students’ professional maturity and growth is reflected through the tasks delegated to them.^26^

Conducive learning environments may assist students to develop their professional dignity. Unfortunately, the participants complained about situations in the hospital that did not contribute to their professional development. An environment where nursing students are regarded as colleagues is conducive to learning and skills development.^21^ An organised, well-resourced clinical learning environment contributes to the professional development of students.^20^

The participants expected that all professional nurses in the hospital would be experts in theory and practice integration. Clinical competency develops when theory is integrated in patient care.^27^ The participants were disappointed in the professional nurses who did not focus on using theory to improve the quality of patient care. Students should be guided to integrate theory in practice during work-integrated learning as it will help them to develop critical clinical decision-making skills.^20^ When theory/practice integration does not take place, nurses’ professional and scientific capabilities get threatened to the detriment of their professional dignity development.^28^

The participants wished to be considered as parts of not only nursing teams, but also multidisciplinary teams. According to them their participation in the teams would have enabled them to develop their professional dignity. In team work, members get acknowledged for their capabilities and more importantly for their positive input to quality patient care.^29^ When students are invited to become team members their professional dignity may be strengthened. Being a part of teams enables students to have a sense of belonging to and being respected by other members of the team.^30^ Nurses’ professional dignity is enhanced when they are respected and their knowledge and skills are appreciated.^28^ The same applies to students. When they are considered as valuable members of health teams their dignity gets enhanced.^5^ Acknowledgement of their professional status by team members may lead them to feel like dignified human beings.^24^ Respect for colleagues, even for student colleagues, is an important value associated with professionalism.^31^ The participants highlighted their need for professional respect from professional nurses. Such respect made them feel dignified.

Nursing students’ learning experience is positively influenced by the support they receive from lecturers.^26^ It reduces their fears and anxieties for the unknown that they associate with the clinical environment.^20^ With the support of their lecturers they feel cared for^24^ and experience feelings of belonging to a caring nursing education institution.^32,33^ Traumatic experiences in the clinical environment such as patients’ deaths may emotionally upset students.^34^ At the same time, academic stressors are experienced. The stress that they experience in both the clinical and academic environment may be to the detriment of their well-being.^35^ It might also impact negatively on their professional growth and dignity.^36^ Effective collaboration between the professional nurses of the clinical and educational institutions to reduce the anxiety that students experience may help them to develop positive self-esteems and develop coping resources to manage stress as professional nurses.^37^ Aligning clinical learning opportunities with theoretical teaching and learning may reduce students’ anxiety when they are expected to perform nursing procedures on patients.^38^ Collaboration between professional nurses in the clinical and educational environments favour the use of learning opportunities for the professional growth of students.^39^ It should be reflected in policies to prevent conflicting practices between staff from both institutions leading to negative learning environments for students.^20^ In their study findings, Allamar, Ahmad, Almutairi and Salem affirm that cooperation between the nursing education institution and clinical practice facilities plays an important role in providing adequate learning opportunities to nursing students to enhance their development into professional nurses.^40^

The significance of the study refers to the contribution that the findings make to the knowledge base of the professional dignity of nurses and the input that supervisors of students during work-integrated learning should make towards their professional development. Student accompaniment should not only focus in enabling them to gain knowledge and to develop skills. It should also help them to grow professionally to become dignified nurses. The study was unfortunately limited to a designated nursing education in South Africa. The researchers hope that it will be repeated it in other institutions and in other countries.

## Conclusions and recommendations

Nursing students develop their professional dignity when they are respected, their contribution to quality patient care is acknowledged and they are appreciated as valuable members of nursing teams. They want the professional nurses who supervise them during work-integrated learning to be positive role models. Students’ professional growth is enhanced when they do clinical work in learning-conducive environments. The study findings highlighted poor supervision and poor guidance as inhibiting aspects of nursing students’ development of professional dignity.

The authors recommend that supportive learning environments be created to ensure a smooth transition of learning from lectures into practice. Efficient communication between the managements of nursing education institutions and hospitals may benefit the students’ professional growth. It may enable them to use the knowledge that they gained at educational institutions, in the delivery of patient care during work-integrated learning. The acknowledgement of their achievements and inclusion in nursing teams may assist them to develop a sense of dignity associated with being nurses. Sufficient time should be spent on the planning and execution of theory and practice integration. The standardisation of clinical procedures is recommended. It may prevent student confusion and experiences of incompetency. Students want professional nurses to demonstrate procedures to them, thereafter to supervise them when they implement it, followed by sufficient opportunities to perform the procedures. Students who successfully perform nursing care, find it easy to grow professionally and to develop a sense of pride in the profession.

## Data Availability

The data that support the findings of the study are available from the corresponding author, upon reasonable request.*
